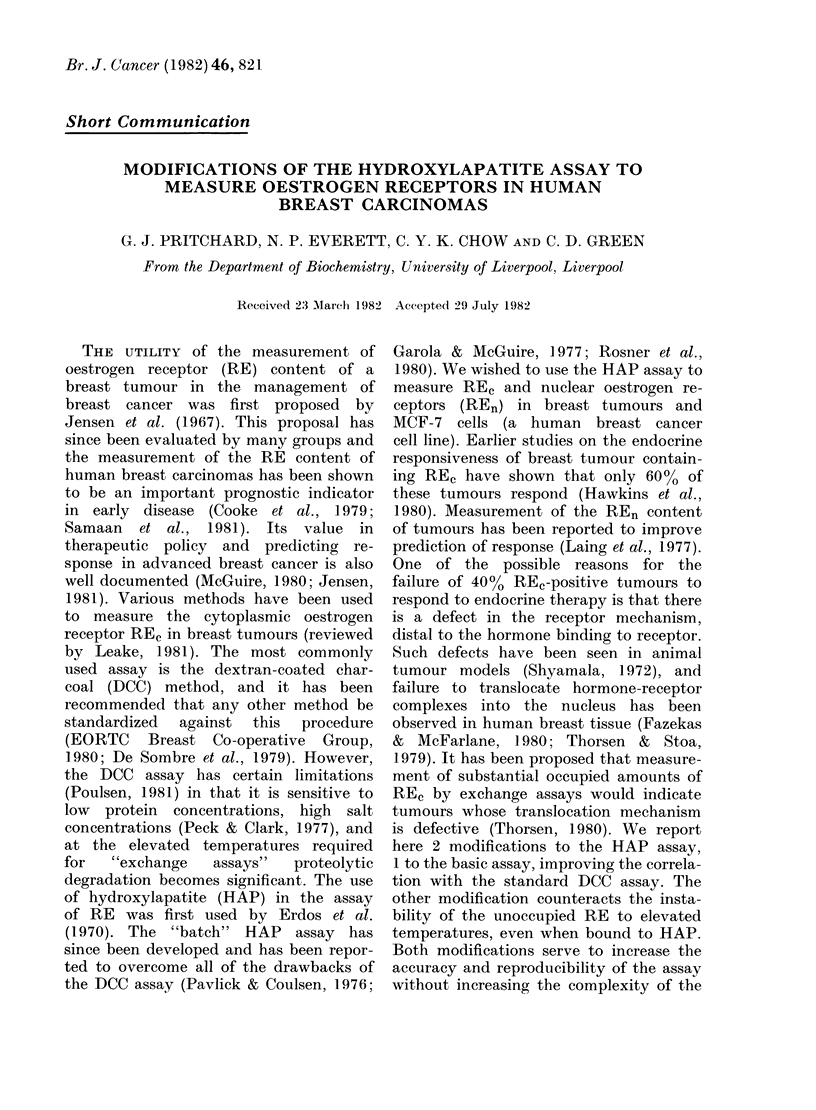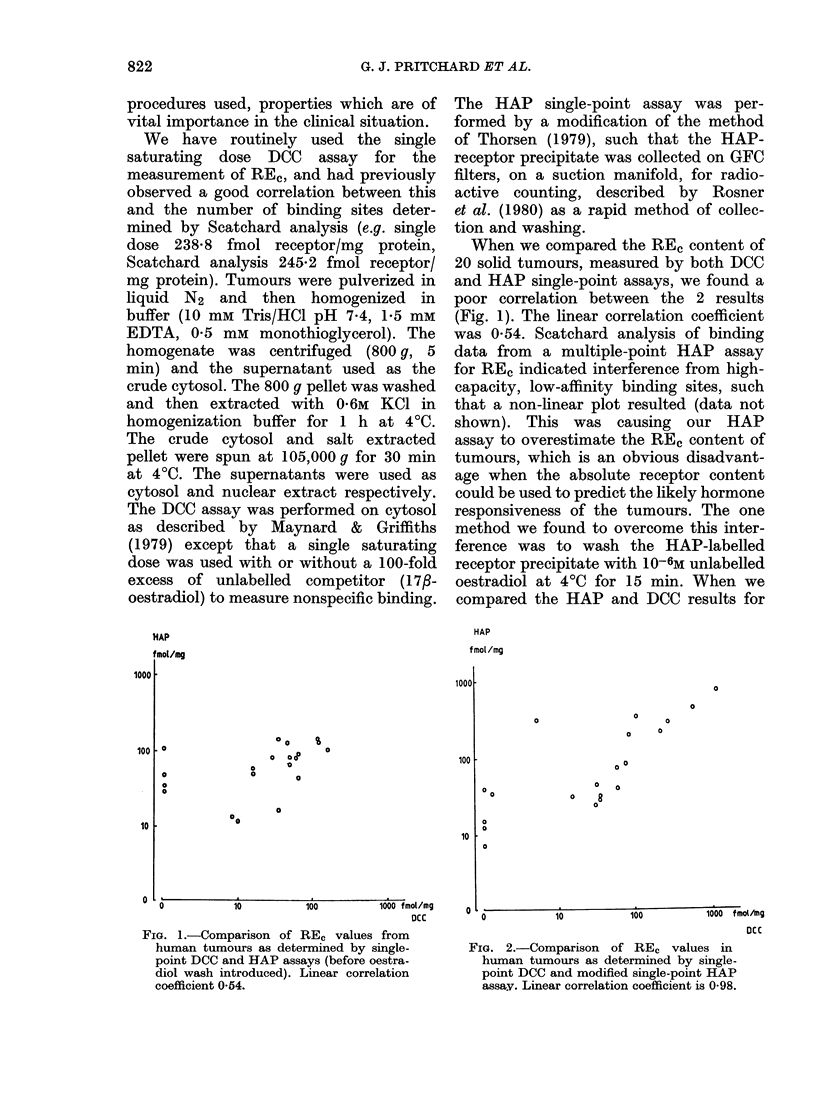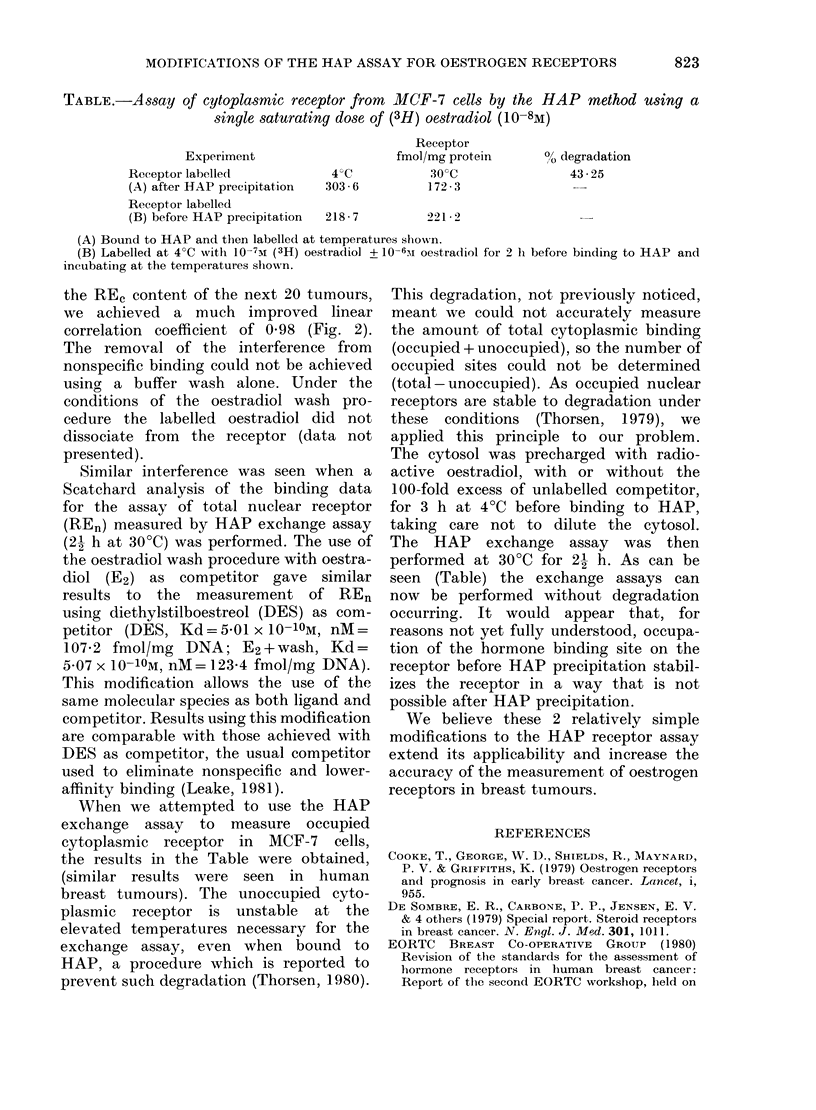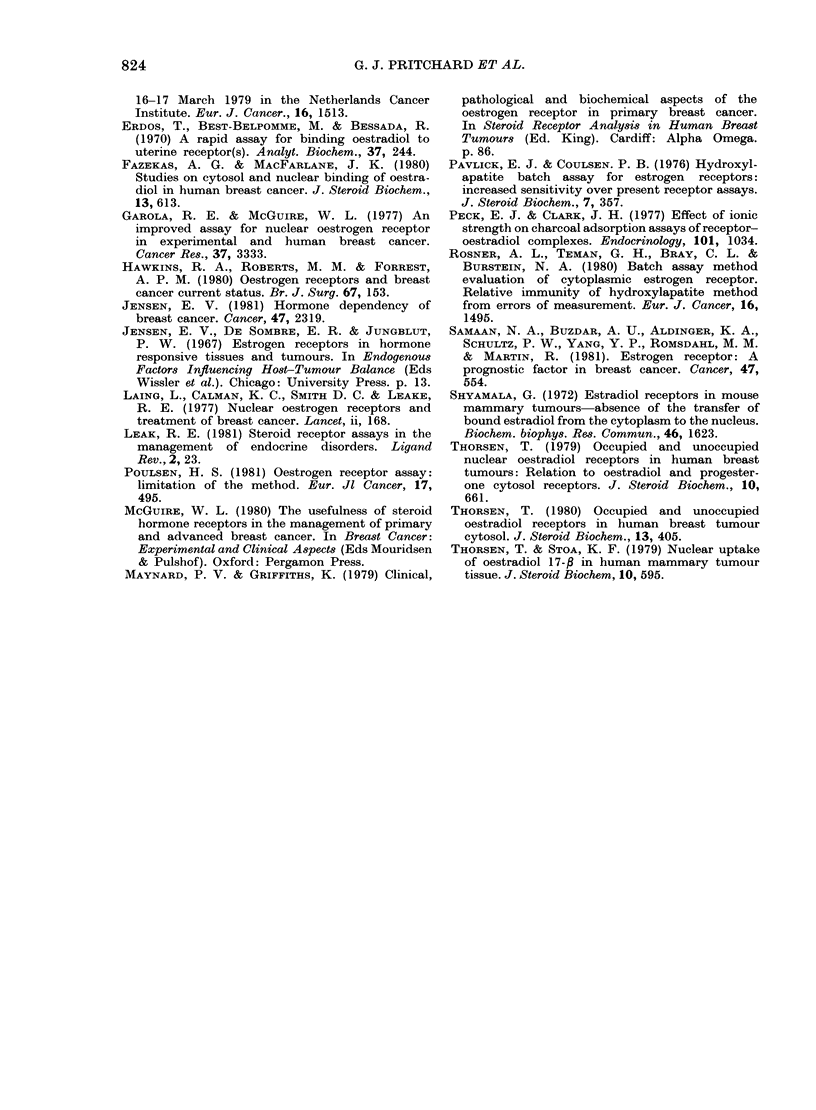# Modifications of the hydroxylapatite assay to measure oestrogen receptors in human breast carcinomas.

**DOI:** 10.1038/bjc.1982.277

**Published:** 1982-11

**Authors:** G. J. Pritchard, N. P. Everett, C. Y. Chow, C. D. Green


Br. J. Cancer (1982) 46, 821

*Short Communication*

### MODIFICATIONS OF THE HYDROXYLAPATITE ASSAY TO MEASURE OESTROGEN RECEPTORS IN HUMAN BREAST CARCINOMAS

G. J. PRITCHARD, N. P. EVERETT, C. Y. K. CHOW AND C. D. GREEN

*From the Department of Biochemistry, University of Liverpool, Liverpool*

Received 23 March 1982 Accepted 29 July 1982

THE UTILITY of the measurement of oestrogen receptor (RE) content of a breast tumour in the management of breast cancer was first proposed by Jensen *et al.* (1967). This proposal has since been evaluated by many groups and the measurement of the RE content of human breast carcinomas has been shown to be an important prognostic indicator in early disease (Cooke *et al.*, 1979; Samaan *et al.*, 1981). Its value in therapeutic policy and predicting response in advanced breast cancer is also well documented (McGuire, 1980; Jensen, 1981). Various methods have been used to measure the cytoplasmic oestrogen receptor $RE_c$ in breast tumours (reviewed by Leake, 1981). The most commonly used assay is the dextran-coated charcoal (DCC) method, and it has been recommended that any other method be standardized against this procedure (EORTC Breast Co-operative Group, 1980; De Sombre *et al.*, 1979). However, the DCC assay has certain limitations (Poulsen, 1981) in that it is sensitive to low protein concentrations, high salt concentrations (Peck & Clark, 1977), and at the elevated temperatures required for "exchange assays" proteolytic degradation becomes significant. The use of hydroxylapatite (HAP) in the assay of RE was first used by Erdos *et al.* (1970). The "batch" HAP assay has since been developed and has been reported to overcome all of the drawbacks of the DCC assay (Pavlick & Coulsen, 1976; Garola & McGuire, 1977; Rosner *et al.*, 1980). We wished to use the HAP assay to measure $RE_c$ and nuclear oestrogen receptors ($RE_n$) in breast tumours and MCF-7 cells (a human breast cancer cell line). Earlier studies on the endocrine responsiveness of breast tumour containing $RE_c$ have shown that only 60% of these tumours respond (Hawkins *et al.*, 1980). Measurement of the $RE_n$ content of tumours has been reported to improve prediction of response (Laing *et al.*, 1977). One of the possible reasons for the failure of 40% $RE_c$-positive tumours to respond to endocrine therapy is that there is a defect in the receptor mechanism, distal to the hormone binding to receptor. Such defects have been seen in animal tumour models (Shyamala, 1972), and failure to translocate hormone-receptor complexes into the nucleus has been observed in human breast tissue (Fazekas & McFarlane, 1980; Thorsen & Stoa, 1979). It has been proposed that measurement of substantial occupied amounts of $RE_c$ by exchange assays would indicate tumours whose translocation mechanism is defective (Thorsen, 1980). We report here 2 modifications to the HAP assay, 1 to the basic assay, improving the correlation with the standard DCC assay. The other modification counteracts the instability of the unoccupied RE to elevated temperatures, even when bound to HAP. Both modifications serve to increase the accuracy and reproducibility of the assay without increasing the complexity of the procedures used, properties which are of vital importance in the clinical situation.

We have routinely used the single saturating dose DCC assay for the measurement of $RE_c$, and had previously observed a good correlation between this and the number of binding sites determined by Scatchard analysis (*e.g.* single dose 238·8 fmol receptor/mg protein, Scatchard analysis 245·2 fmol receptor/mg protein). Tumours were pulverized in liquid $N_2$ and then homogenized in buffer (10 mM Tris/HCl pH 7·4, 1·5 mM EDTA, 0·5 mM monothioglycerol). The homogenate was centrifuged (800 *g*, 5 min) and the supernatant used as the crude cytosol. The 800 *g* pellet was washed and then extracted with 0·6M KCl in homogenization buffer for 1 h at 4°C. The crude cytosol and salt extracted pellet were spun at 105,000 *g* for 30 min at 4°C. The supernatants were used as cytosol and nuclear extract respectively. The DCC assay was performed on cytosol as described by Maynard & Griffiths (1979) except that a single saturating dose was used with or without a 100-fold excess of unlabelled competitor (17β-oestradiol) to measure nonspecific binding. The HAP single-point assay was performed by a modification of the method of Thorsen (1979), such that the HAP-receptor precipitate was collected on GFC filters, on a suction manifold, for radioactive counting, described by Rosner *et al.* (1980) as a rapid method of collection and washing.

When we compared the $RE_c$ content of 20 solid tumours, measured by both DCC and HAP single-point assays, we found a poor correlation between the 2 results (Fig. 1). The linear correlation coefficient was 0·54. Scatchard analysis of binding data from a multiple-point HAP assay for $RE_c$ indicated interference from high-capacity, low-affinity binding sites, such that a non-linear plot resulted (data not shown). This was causing our HAP assay to overestimate the $RE_c$ content of tumours, which is an obvious disadvantage when the absolute receptor content could be used to predict the likely hormone responsiveness of the tumours. The one method we found to overcome this interference was to wash the HAP-labelled receptor precipitate with $10^{-6}$M unlabelled oestradiol at 4°C for 15 min. When we compared the HAP and DCC results for

FIG. 1.—Comparison of $RE_c$ values from human tumours as determined by single-point DCC and HAP assays (before oestradiol wash introduced). Linear correlation coefficient 0·54.

FIG. 2.—Comparison of $RE_c$ values in human tumours as determined by single-point DCC and modified single-point HAP assay. Linear correlation coefficient is 0·98.

TABLE.—*Assay of cytoplasmic receptor from MCF-7 cells by the HAP method using a single saturating dose of* ($^3H$) *oestradiol* ($10^{-8}$M)

| Experiment | | Receptor fmol/mg protein | % degradation |
|---|---|---|---|
| Receptor labelled | 4°C | 30°C | 43·25 |
| (A) after HAP precipitation | 303·6 | 172·3 | — |
| Receptor labelled | | | |
| (B) before HAP precipitation | 218·7 | 221·2 | — |

(A) Bound to HAP and then labelled at temperatures shown.

(B) Labelled at 4°C with $10^{-7}$M ($^3$H) oestradiol $\pm 10^{-6}$M oestradiol for 2 h before binding to HAP and incubating at the temperatures shown.

the $RE_c$ content of the next 20 tumours, we achieved a much improved linear correlation coefficient of 0·98 (Fig. 2). The removal of the interference from nonspecific binding could not be achieved using a buffer wash alone. Under the conditions of the oestradiol wash procedure the labelled oestradiol did not dissociate from the receptor (data not presented).

Similar interference was seen when a Scatchard analysis of the binding data for the assay of total nuclear receptor ($RE_n$) measured by HAP exchange assay ($2\frac{1}{2}$ h at 30°C) was performed. The use of the oestradiol wash procedure with oestradiol ($E_2$) as competitor gave similar results to the measurement of $RE_n$ using diethylstilboestreol (DES) as competitor (DES, Kd = $5{\cdot}01 \times 10^{-10}$M, nM = 107·2 fmol/mg DNA; $E_2$ + wash, Kd = $5{\cdot}07 \times 10^{-10}$M, nM = 123·4 fmol/mg DNA). This modification allows the use of the same molecular species as both ligand and competitor. Results using this modification are comparable with those achieved with DES as competitor, the usual competitor used to eliminate nonspecific and lower-affinity binding (Leake, 1981).

When we attempted to use the HAP exchange assay to measure occupied cytoplasmic receptor in MCF-7 cells, the results in the Table were obtained, (similar results were seen in human breast tumours). The unoccupied cytoplasmic receptor is unstable at the elevated temperatures necessary for the exchange assay, even when bound to HAP, a procedure which is reported to prevent such degradation (Thorsen, 1980). This degradation, not previously noticed, meant we could not accurately measure the amount of total cytoplasmic binding (occupied + unoccupied), so the number of occupied sites could not be determined (total − unoccupied). As occupied nuclear receptors are stable to degradation under these conditions (Thorsen, 1979), we applied this principle to our problem. The cytosol was precharged with radioactive oestradiol, with or without the 100-fold excess of unlabelled competitor, for 3 h at 4°C before binding to HAP, taking care not to dilute the cytosol. The HAP exchange assay was then performed at 30°C for $2\frac{1}{2}$ h. As can be seen (Table) the exchange assays can now be performed without degradation occurring. It would appear that, for reasons not yet fully understood, occupation of the hormone binding site on the receptor before HAP precipitation stabilizes the receptor in a way that is not possible after HAP precipitation.

We believe these 2 relatively simple modifications to the HAP receptor assay extend its applicability and increase the accuracy of the measurement of oestrogen receptors in breast tumours.

### REFERENCES

COOKE, T., GEORGE, W. D., SHIELDS, R., MAYNARD, P. V. & GRIFFITHS, K. (1979) Oestrogen receptors and prognosis in early breast cancer. *Lancet*, i, 955.

DE SOMBRE, E. R., CARBONE, P. P., JENSEN, E. V. & 4 others (1979) Special report. Steroid receptors in breast cancer. *N. Engl. J. Med.* **301,** 1011.

EORTC BREAST CO-OPERATIVE GROUP (1980) Revision of the standards for the assessment of hormone receptors in human breast cancer: Report of the second EORTC workshop, held on

16–17 March 1979 in the Netherlands Cancer Institute. *Eur. J. Cancer.*, **16**, 1513.

ERDOS, T., BEST-BELPOMME, M. & BESSADA, R. (1970) A rapid assay for binding oestradiol to uterine receptor(s). *Analyt. Biochem.*, **37**, 244.

FAZEKAS, A. G. & MACFARLANE, J. K. (1980) Studies on cytosol and nuclear binding of oestradiol in human breast cancer. *J. Steroid Biochem.*, **13**, 613.

GAROLA, R. E. & MCGUIRE, W. L. (1977) An improved assay for nuclear oestrogen receptor in experimental and human breast cancer. *Cancer Res.*, **37**, 3333.

HAWKINS, R. A., ROBERTS, M. M. & FORREST, A. P. M. (1980) Oestrogen receptors and breast cancer current status. *Br. J. Surg.* **67**, 153.

JENSEN, E. V. (1981) Hormone dependency of breast cancer. *Cancer*, **47**, 2319.

JENSEN, E. V., DE SOMBRE, E. R. & JUNGBLUT, P. W. (1967) Estrogen receptors in hormone responsive tissues and tumours. In *Endogenous Factors Influencing Host–Tumour Balance* (Eds Wissler *et al.*). Chicago: University Press. p. 13.

LAING, L., CALMAN, K. C., SMITH D. C. & LEAKE, R. E. (1977) Nuclear oestrogen receptors and treatment of breast cancer. *Lancet*, ii, 168.

LEAK, R. E. (1981) Steroid receptor assays in the management of endocrine disorders. *Ligand Rev.*, **2**, 23.

POULSEN, H. S. (1981) Oestrogen receptor assay: limitation of the method. *Eur. Jl Cancer*, **17**, 495.

MCGUIRE, W. L. (1980) The usefulness of steroid hormone receptors in the management of primary and advanced breast cancer. In *Breast Cancer: Experimental and Clinical Aspects* (Eds Mouridsen & Pulshof). Oxford: Pergamon Press.

MAYNARD, P. V. & GRIFFITHS, K. (1979) Clinical, pathological and biochemical aspects of the oestrogen receptor in primary breast cancer. In *Steroid Receptor Analysis in Human Breast Tumours* (Ed. King). Cardiff: Alpha Omega. p. 86.

PAVLICK, E. J. & COULSEN. P. B. (1976) Hydroxylapatite batch assay for estrogen receptors: increased sensitivity over present receptor assays. *J. Steroid Biochem.*, **7**, 357.

PECK, E. J. & CLARK, J. H. (1977) Effect of ionic strength on charcoal adsorption assays of receptor–oestradiol complexes. *Endocrinology*, **101**, 1034.

ROSNER, A. L., TEMAN, G. H., BRAY, C. L. & BURSTEIN, N. A. (1980) Batch assay method evaluation of cytoplasmic estrogen receptor. Relative immunity of hydroxylapatite method from errors of measurement. *Eur. J. Cancer*, **16**, 1495.

SAMAAN, N. A., BUZDAR, A. U., ALDINGER, K. A., SCHULTZ, P. W., YANG, Y. P., ROMSDAHL, M. M. & MARTIN, R. (1981). Estrogen receptor: A prognostic factor in breast cancer. *Cancer*, **47**, 554.

SHYAMALA, G. (1972) Estradiol receptors in mouse mammary tumours—absence of the transfer of bound estradiol from the cytoplasm to the nucleus. *Biochem. biophys. Res. Commun.*, **46**, 1623.

THORSEN, T. (1979) Occupied and unoccupied nuclear oestradiol receptors in human breast tumours: Relation to oestradiol and progesterone cytosol receptors. *J. Steroid Biochem.*, **10**, 661.

THORSEN, T. (1980) Occupied and unoccupied oestradiol receptors in human breast tumour cytosol. *J. Steroid Biochem.*, **13**, 405.

THORSEN, T. & STOA, K. F. (1979) Nuclear uptake of oestradiol 17-$\beta$ in human mammary tumour tissue. *J. Steroid Biochem*, **10**, 595.